# Natural infection of common cranes (*Grus grus*) with highly pathogenic avian influenza H5N1 in Serbia

**DOI:** 10.3389/fvets.2024.1462546

**Published:** 2024-12-09

**Authors:** Biljana Djurdjević, Tamaš Petrović, Vladimir Gajdov, Dejan Vidanović, Ivana Vučićević, Milena Samojlović, Marko Pajić

**Affiliations:** ^1^Scientific Veterinary Institute “Novi Sad”, Novi Sad, Serbia; ^2^Specialized Veterinary Institute “Kraljevo”, Kraljevo, Serbia; ^3^Faculty of Veterinary Medicine, University of Belgrade, Belgrade, Serbia

**Keywords:** avian influenza, common cranes, H5N1, pathology, Serbia

## Abstract

**Introduction:**

The late autumn epizootic of the highly pathogenic avian influenza virus (HPAIV) subtype H5N1 in Serbia in 2023 caused massive mortality in the migratory population of common cranes (*Grus Grus*). This is the first time HPAIV has been identified in the common crane in Serbia, leading to mass mortality of this bird species.

**Methods:**

To understand the pathological impact of HPAIV in cranes, we evaluated the pathological changes in the tissues of common cranes. Additionally, we report genomic characterization of HPAI/H5N1. In total, 14 juvenile common crane carcasses were examined.

**Results:**

Infected birds primarily exhibited neurologic signs, including ataxia and incoordination. Grossly, necrotizing pancreatitis was the most common finding, while microscopic lesions included necrosis, inflammation and hemorrhages in the lungs, spleen, brain, liver and kidneys. Based on RT-PCR, all birds were infected with the HPAI H5N1 virus, as viral RNA was detected in all 14 selected tissues. Genetic analysis revealed that our H5N1 isolate could be grouped with highly pathogenic avian influenza clade 2.3.4.4b, subgroup DA, and is very closely related to the H5N1 strains isolated from the common crane and turkey from Croatia, the common crane from Italy and the Ural owl from Slovakia.

**Discussion:**

Our findings showed that common cranes are highly susceptible to natural infection with the HPAI H5N1 virus of clade 2.3.4.4b and may serve as bio-sentinels for the presence of the HPAI virus in wildlife.

## 1 Introduction

Since its emergence over 20 years ago in Asia, avian influenza caused by A(H5Nx) subtype viruses in the goose/Guangdong/1/96 lineage (gsGD) has undergone extensive genetic diversification including the formation of hundreds of genotypes following reassortment with other avian influenza A viruses (1). The initial outbreaks of highly pathogenic avian influenza (HPAI) H5 subtype viruses were mainly reported in the poultry population. However, since 2005, numerous outbreaks among different wild birds have been reported around the globe, becoming a global public health concern (2). From late 2005 to early 2006, HPAI (H5N1) was detected for the first time in birds in Eastern Europe, the Middle East, and Northern Africa, suggesting that the virus spread through wild bird migration (3). Consequently, Serbia reported the first outbreak of HPAI H5N1 in poultry and mute swans in various regions in 2006 (4). Ten years later, in the winter of 2016/2017, new HPAI cases were detected and since then, sporadic cases of HPAI virus infection have been reported almost every year in the Republic of Serbia, affecting both wild bird population and backyard poultry. Most cases have been detected in the northern part of the country, in the Province of Vojvodina (5). The HPAI H5N8 epidemic in Serbia during 2016/2017 caused massive wild bird mortality, primarily affecting mute swans (*Cygnus Olor*) (6). Similarly, during the previous epidemic in 2021/2022, a related H5N8 virus of clade 2.3.4.4b caused moderate mortality among wild birds, predominantly affecting mute swans.

However, during the current HPAI H5N1 epidemic in Serbia in 2023/2024, only a limited number of swans were found dead, suggesting differences in the pathogenicity of these two clades 2.3.4.4b HPAI viruses for certain bird species. However, one new bird species that proved to be highly susceptible during the current epidemic was the common crane (*Grus grus*). Overwintering areas for cranes in Serbia are located in the northern part of the country, in the Province of Vojvodina, and the most important overwintering area is the special nature reserve “Slano Kopovo.” This special nature reserve is critically important as a habitat for migratory bird species and represents a stopover for cranes on their migration route from northern Europe where they breed to Africa. During autumn, this location can host up to 20,000 cranes (7), often congregating alongside several thousand other birds, primarily geese and ducks. The main factors driving such large aggregations include the availability of food and the region’s advantageous geographical position. While the high density of birds and associated risk factors may heighten the potential for an HPAI outbreak, it is noteworthy that HPAI infections had not been reported in this area prior to the recent outbreak.

In late November and early December 2023, a mass mortality of common cranes was reported in several locations in the Province of Vojvodina, mostly in above mentioned special nature reserve “Slano Kopovo.” The increased alertness of ornithologists for dead and diseased cranes during this HPAIV epizootic led to the identification and reporting of suspect cases to local veterinary authorities. According to field veterinarians and veterinary inspectors, more than several hundred carcasses were found in these locations during this period (estimated of more than 500).

Considering that this is the first detection of HPAI virus in this bird species in Serbia, our research aimed to compare pathological lesions in cranes affected by this fatal disease, to those observed in other avian species. We tested all cranes brought to Scientific Veterinary Institute “Novi Sad” during the autumn/winter HPAI outbreak in 2023, conducting full necropsies of these birds to identify HPAI-associated lesions. To determine the possible origin of these viral isolates, phylogenetic analysis and genetic characterization of the virus were conducted. This paper combines field observations, pathological examinations, and molecular analyses to investigate the various factors that contributed to the high mortality rates among common cranes in late autumn 2023.

## 2 Materials and methods

**Figure 1:**
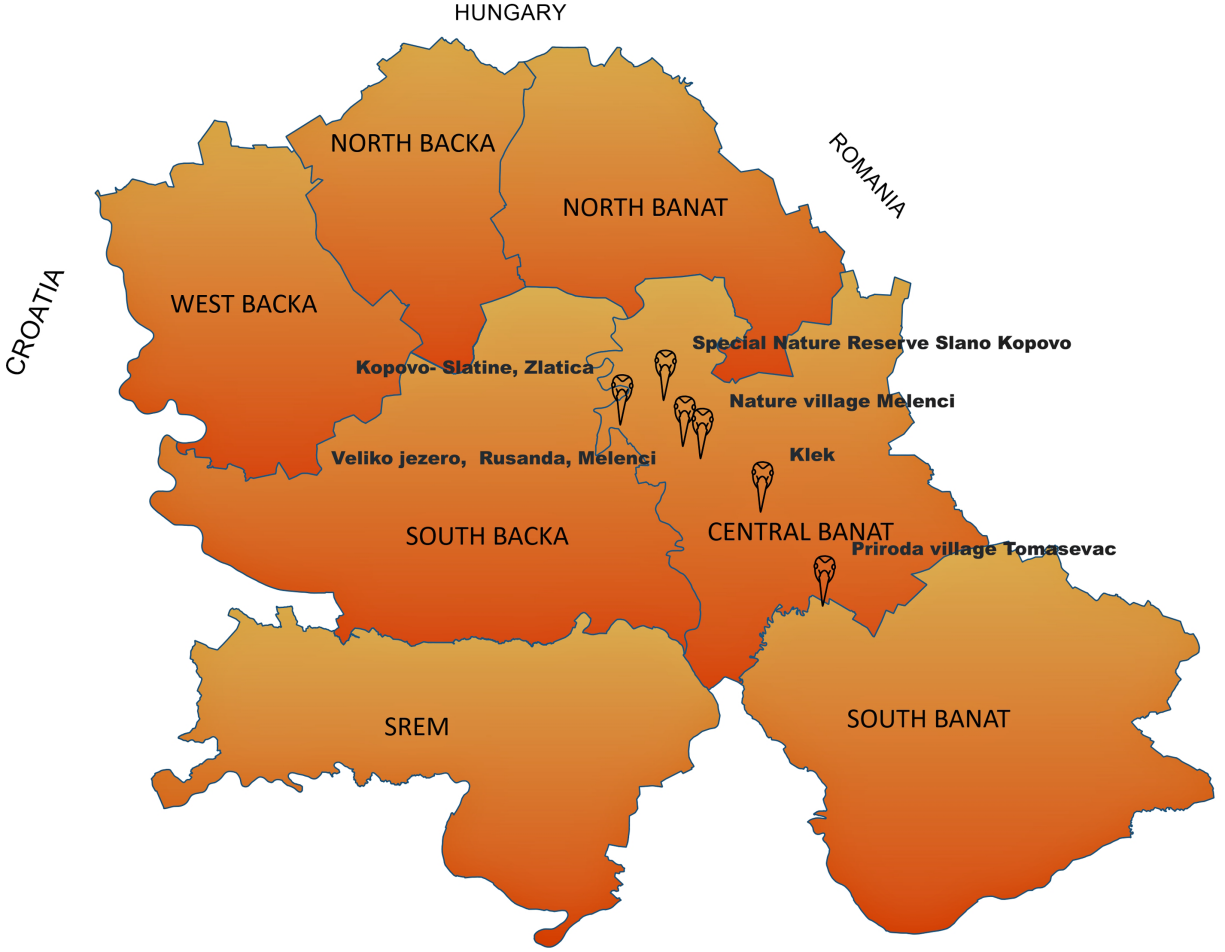
Positive cases of HPAI H5N1 detected in common cranes in the Province of Vojvodina during autumn/winter epizootic in 2023 (map shows the locations of bird samples collected in this study).

In total, 14 common crane carcasses were collected in late November and early beginning of the December 2024 in the field and submitted to the Scientific Veterinary Institute “Novi Sad” to determine the cause of death. These specimens were collected as part of the Serbian avian influenza wild bird passive surveillance monitoring. All carcasses were collected in Central Banat District:—five carcasses were collected from the special nature reserve “Slano Kopovo”; five carcasses were collected from the nature reserve “Slatine”; one carcass was collected from Lake Rusanda; one from the suburban area Tomaševac; one from Melenci field; and one from a fishpond near Klek village (Figure 1). The carcasses underwent postmortem evaluation prior to delivery, including gross pathology and histopathology. Tissue samples (brain, kidney, spleen, lung, pancreas, and liver) were fixed in 10% neutral buffered formalin for at least 72 h, routinely processed for histopathology, and embedded in paraffin. The paraffin-embedded tissues were sectioned into 4–5 μm-thick sections and stained with hematoxylin and eosin (H&E). The tissues were assessed using a conventional light microscope for histopathology (Zeiss, Germany), and lesions were scored as absent, mild, moderate, or severe.

### 2.1 Virological and phylogenetic investigation

Tissue samples of the brain, pancreas, liver, kidney, and lungs from crane carcasses were pooled for each bird and tested for the presence of HPAI virus using real-time reverse transcription PCR (RT-qPCR) for the presence of HPAI virus. Briefly, tissue samples were cut into small pieces weighing 0.2 g and placed in 2 mL micro tubes and homogenized in 1 mL sterile phosphate-buffered saline for 5 min using a TissueLyser LT (Qiagen, Hilden, Germany) operating at 50 Hz. The homogenates were centrifuged for 10 min at 2,000 g. Supernatant was used for RNA extraction. Total RNA was extracted using the commercial IndiSpin Pathogen Kit (Indical Bioscience GmbH, Germany) according to the manufacturer’s instructions. The detection of matrix gen (M gene), suitable for detection of all influenza viruses, and detection of H5 gene was performed by TaqMan-based one-step RT-qPCR with oligonucleotide primers and probes and thermal profiles described by Spackman et al. (8), and by using commercial kit RNA UltraSense™ One-Step Quantitative RT-PCR System (Invitrogen, ThermoFisher Scientific), according to the manufacturer’s instructions. The N1 gene was detected by TaqMan-based one-step RT-qPCR with oligonucleotide primers and probe and thermal profile as described by Hoffman et al. (9), and by using the same, previously described, commercial one-step RT-qPCR kit.

For differential diagnostic purposes, tissue samples from common crane carcasses were also examined for the presence of Avian orthoavulavirus 1 (AOaV-1), previously known as Avian paramyxovirus 1—APMV-1. AOaV-1 was detected using the same methodology (TaqMan-based one-step RT-qPCR) and chemistry as it was used for the detection of AIV, and with oligonucleotide primers and probe and thermal profile as described by Wise et al. (10).

Next-generation sequencing was performed directly from clinical material using amplification protocol described (11). Library preparation was performed using Ligation Sequencing Kit (SQK-LSK109, ONT) and sequencing was performed using R.9.4.1 flow cell (FLO-MIN106D, Oxford Nanopore Technologies) on Oxford Nanopore MinION device. Consensus sequence was obtained using Minimap 2 mapper on Geneious prime software (v2023). The evolutionary history was inferred by using the Maximum Likelihood method and the Tamura-Nei model (12). Evolutionary analyses were conducted in MEGA X (13). The dataset generated in this study is available in GISAID under the accession number EPI _ISL_19176354.

## 3 Results

### 3.1 Pathological findings

The clinical disease reported in common cranes included specific neurological signs such as twitching, ataxia, incoordination, lethargy, and sudden death. Clinical history was obtained from ornithologists in the field and through veterinary inspection via live observation. All examined birds were in good body condition, with abundant subcutaneous fat and fat in the body cavity and had well-developed pectoral muscles. All carcasses were fresh, except for one that was moderately fresh. All animals were juvenile, as indicated by the presence of thymus glands. The ratio of males to females was equal. No visible external lesions or injuries were detected.

**Figure 2:**
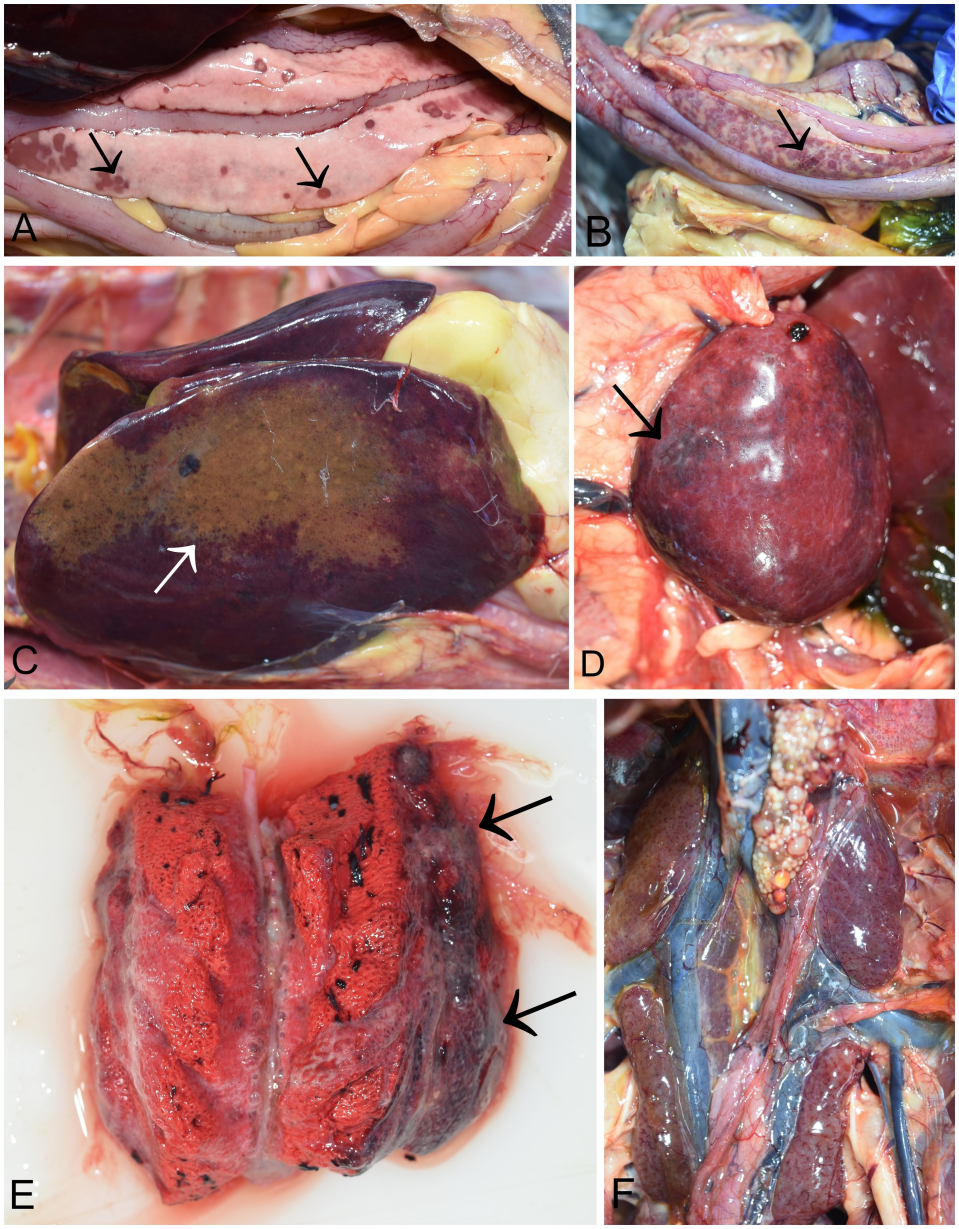
Necropsy findings in common cranes (*Grus grus*) naturally infected with H5N1 HPAIV. **(A)** Pancreas of a common crane showing various size of dark necrotic foci (arrow). **(B)** Hemorrhages in the pancreas (arrow); **(C)** Liver, diffuse necrotic foci **(D)** Spleen, diffusely enlarged and mottled with hemorrhages (arrow); **(E)** Lung, coalescing pulmonary consolidation with marked dark red and firm areas (arrows). **(F)** Kidneys, mild petechial hemorrhages.

At necropsy, the pancreas was severely affected in all birds (14/14). The most consistent and predominant lesions were multifocal, demarcated, and partly coalescent necrosis. Necrotic foci varied in size and were white or dark pink (Figure 2A). Pancreatic petechial hemorrhages were observed in 10 cases (10/14) (Figure 2B). In four cases, diffusely yellow necrotic foci were noted in the liver (Figure 2C), along with an enlarged and swollen liver with rounded margins. A moderately enlarged and congested spleen was characteristic, though not present in all infected birds. (Figure 2D). Moderate to severe unilateral or bilateral lung congestion were recorded in five cases. Occasionally, the lungs were diffusely edematous and mottled from pink to dark red (Figure 2E). In two cases, increased pericardial hemorrhagic fluid was also observed. Petechial hemorrhages in kidneys were seen in five cases (Figure 2F). Hyperemia of cerebral blood vessels were present in three cases. All birds had scarce food remains in the gizzard. However, no visible lesions were detected in the proventriculus, gizzard, or intestines. The other organs appeared grossly normal.

**Figure 3:**
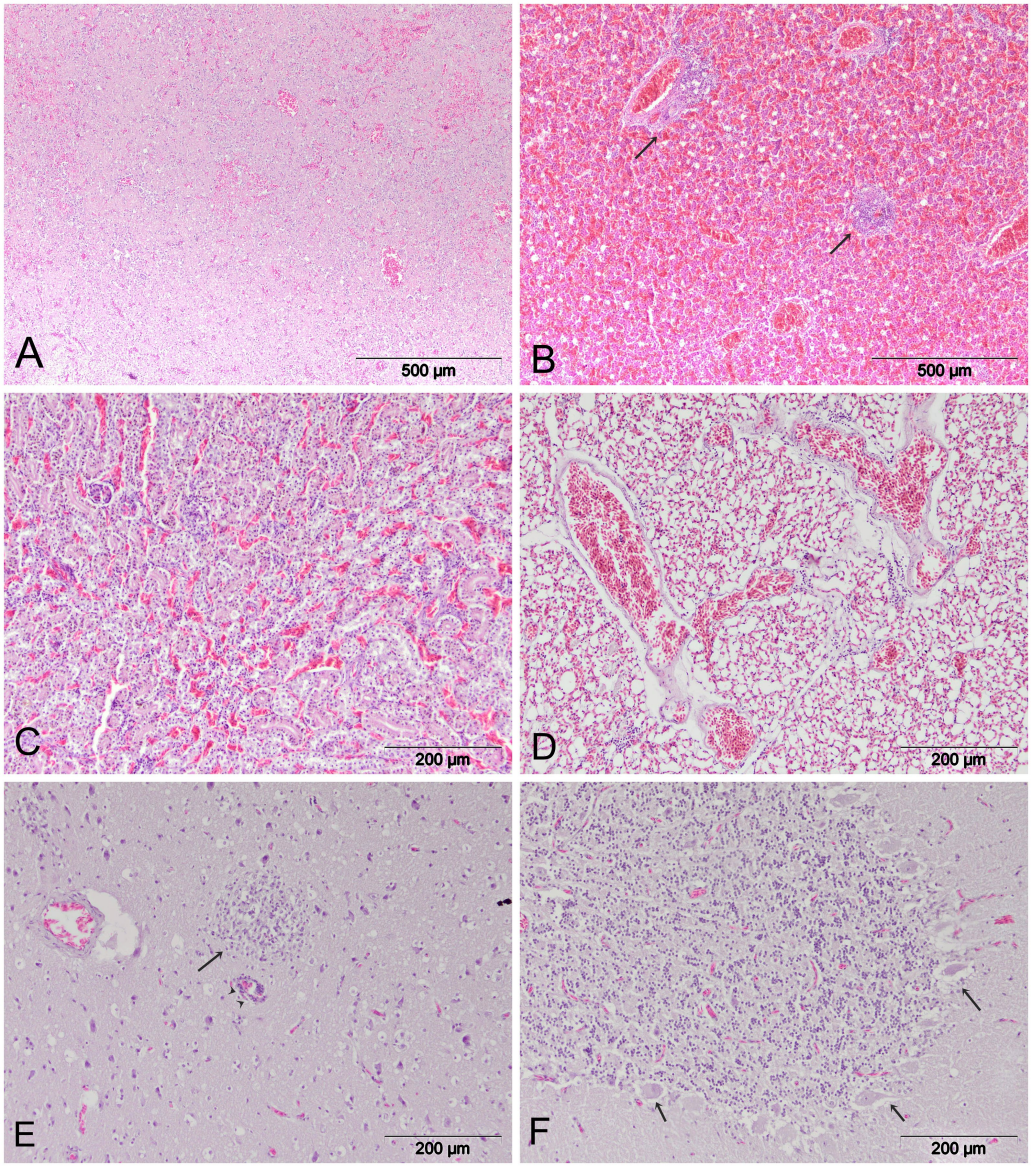
Histopathologic lesions in common cranes naturally infected with HPAI H5N1. **(A)** Pancreas. Confluent necrosis and diffuse hemorrhages. **(B)** Liver. Multifocal hemorrhages and mononuclear infiltration (arrow). **(C)** Kidney. Tubulonecrosis and hemmorhages. **(D)** Lungs. Diffuse hemorrhages and generalized congestion; **(E)** Brain. Non-purulent encephalitis (arrow), inflammatory perivascular cuffing (arrowhead) and mild neuropil edema. **(F)** Cerebellum. Degeneration of Purkinje cells.

Microscopically, all examined organs showed moderate to severe histological lesions (Figure 3). The pancreas was particularly severely affected, with moderate to severe areas of confluent necrosis of acinar cells and marked infiltration by mononuclear cells. Severe hemmorrhages were present in most examined cases (Figure 3A). In the liver, there was random mild to moderate hepatocellular necrosis. Additional lesions regularly observed in the liver included hemorrhages and multifocal perivascular cuffing (Figure 3B). Most of the birds showed mild interstitial nephritis, tubulonecrosis, and severe hemorrhages in the kidneys (Figure 3C). The lungs exhibited diffuse vascular congestion and diffuse hemorrhages (Figure 3D). In the spleen, there were findings of lymphoid depletion, multifocal necrosis and hemmorrhages. Brain lesions were multifocal and randomly distributed, with all birds showing multifocal lymphocytic meningoencephalitis. Furthermore, mild to moderate multifocal mononuclear cell infiltration and perivascular mononuclear cells were observed in the brain of six cranes. Mild neuropil edema was present in all cases (Figure 3E). Multifocal encephalomalacia, foci of gliosis and neuronal degeneration were frequently observed. In the cerebellum, there was degeneration of Purkinje cells with surrounding edema (Figure 3F).

The summary of gross and histopathological findings is detailed in Supplementary Table 1.

### 3.2 Virology

The presence of matrix gene of influenza viruses was detected in tissue samples of all out of 14 individual cranes tested animals by RT-qPCR method. The virus load in the samples was very high, with Ct values ranging from 16 and 25. In addition, the presence of H5 and N1 gene (H5N1 virus subtype) of influenza virus was confirmed in all influenza virus positive common crane samples collected in different localities by using the RT-qPCR method for subtyping. The presence of the AOaV-1 virus was not detected in any of the tested samples, originating from 14 dead common cranes.

**Figure 4:**
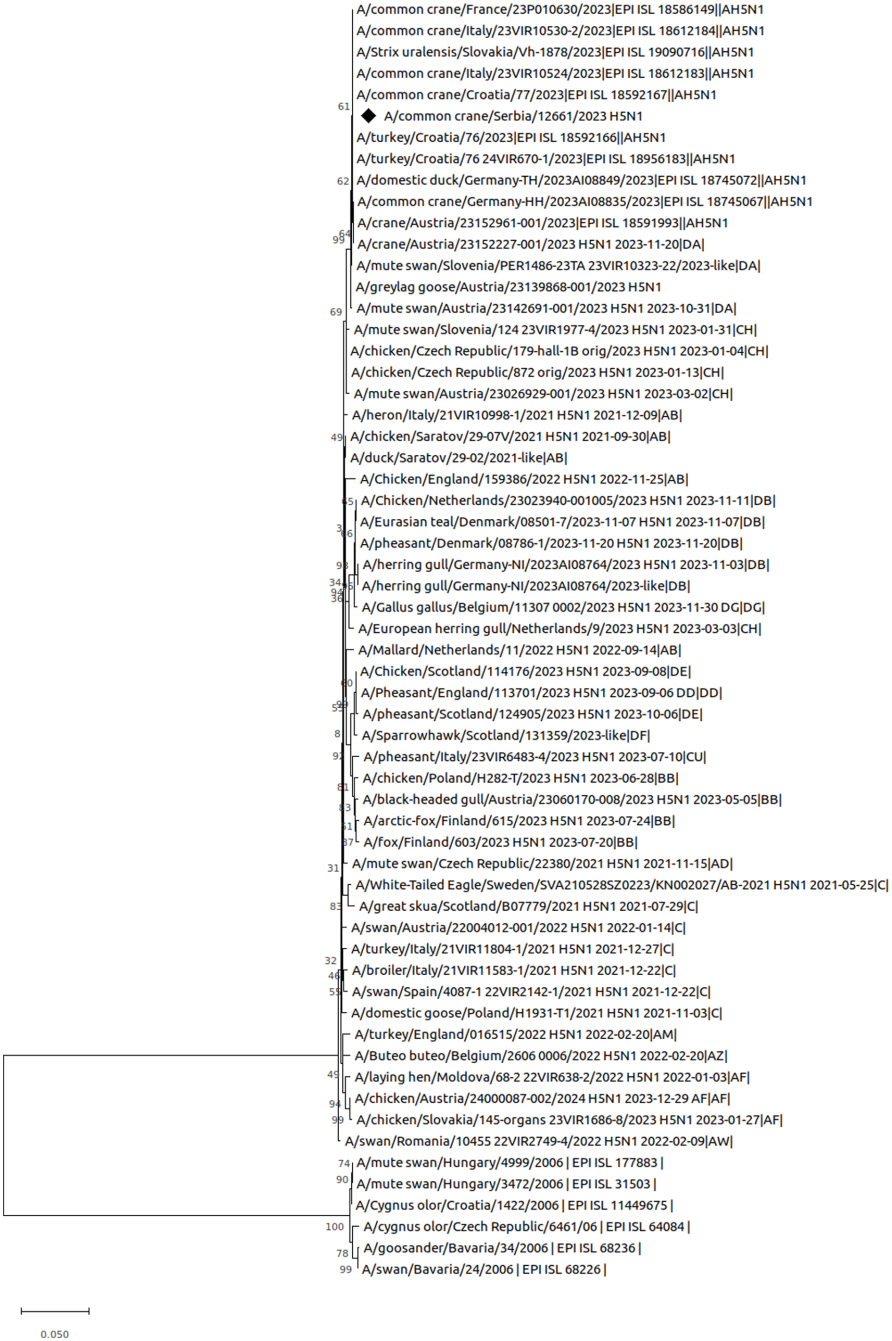
Evolutionary analysis by Maximum Likelihood method. The tree with the highest log likelihood (−6168.83) is shown. The percentage of trees in which the associated taxa clustered together is shown next to the branches. Initial tree(s) for the heuristic search were obtained automatically by applying Neighbor-Join and BioNJ algorithms to a matrix of pairwise distances estimated using the Tamura-Nei model, and then selecting the topology with superior log likelihood value. This analysis involved 60 nucleotide sequences. Codon positions included were 1st + 2nd + 3rd + Noncoding. There were a total of 1702 positions in the final dataset.

To find genetically most closely related strains, we performed a search within the Global Initiative on Sharing Avian Influenza Data (GISAID) database. Based on phylogenetic reconstruction of HA gene, our results showed that our H5N1 isolate from common crane can be grouped with highly pathogenic avian influenza clade 2.3.4.4b, subgroup DA, and that is very closely related to the H5N1 strains isolated from common crane and turkey from Croatia (A/common crane/Croatia/77/2023, A/turkey/Croatia/76/2023, A/turkey/Croatia/76 24VIR670-1/2023), common crane from Italy (A/common crane/Italy/23VIR10524/2023), and Ural owl (*Strix Uralensis*) from Slovakia (A/*Strix uralensis*/Slovakia/Vh-1878/2023) (Figure 4).

## 4 Discussion

This study details an occurrence of massive mortality from HPAI in common cranes (*G. grus*) in Serbia. In contrast to previous HPAI outbreaks in wild birds in Serbia (6, 14), this autumn/winter epidemic shows lower mortality rate in mute swans, while cranes were the most affected bird species. The common cranes are fully migratory species with worldwide distribution (15). Locations in the Province of Vojvodina where deaths of this species were recorded are considered as the main wintering sites for the common cranes in Serbia. In contrast to research from China in 2021 (16), in which the HPAI H5N8 virus was detected in cranes and in which cranes were found to be asymptomatic carriers of the virus, the autumn/winter outbreak of H5N1 in 2023 led to a mass death of cranes in several European countries, including Serbia (17). This is the first time that HPAI has been recorded to cause high-level mortality in common cranes in Europe. The only previously known die-off in common cranes from HPAI was in Israel in autumn 2021, when an estimated 10,000 common cranes on their migration along the East European route from Russia and Scandinavia to Ethiopia and Sudan died from HPAI H5N8 virus infection in and around the Hula Valley in Israel (18).

According to the clinical signs and character of the pathologic lesions, it can be concluded that the disease took as per acute and acute course. Clinically, although birds showed specific neurological signs, there was no ocular involvement, such as corneal opacity, as previously observed in HPAI H5N1-infected ducks (19, 20) Further, all examined individuals were juvenile, which potentially indicates the sensitivity of this crane age category to HPAI H5N1. Severe diffuse hemorrhages in serosal surfaces of internal organs, subcutaneous tissue, coronary and coelomic fat and skeletal muscles, as previously described in HPAI-infected mute swans (6, 14), were absent.

In this bird species, H5N1 showed a tropism for pancreatic tissue, comparable to that observed in most other susceptible wild bird species (21–23). This investigation of naturally acquired infection in cranes revealed that gross pathology in all birds was limited to lesions of the pancreas, with occasional lesions in other organs. The character of the pancreatic lesions in cranes corresponds with those seen previously in H5N1-infected cranes (18) and other avian species, including naturally and experimentally H5N1-infected ones (24–26). Except for the described pancreatic lesions, gross lesions in other organs were mild to moderate and were detected sporadically. These findings, along with the clear lack of gross pathological changes that can be considered strongly pathognomonic for HPAIV, demonstrate the difficulty in diagnosing AI infection among cranes by gross pathology examination alone. These findings also imply that HPAI virus infections in cranes may be overlooked during routine necropsy. However, in suspected cases, the pancreas must always be examined in detail during bird necropsies. Additionally, H5N1 in these common cranes was clearly neurotropic and associated with meningoencephalitis, similar to findings in naturally infected wild birds (14, 21, 27, 28).

Phylogenetic analyses indicated a close relationship between the virus and H5N1 strains isolated from common cranes in Croatia and Italy. This suggests that the most likely source of infection in cranes in Serbia was the introduction of migratory cranes that transmitted the disease. During the wintering season, cranes typically congregate in high densities for feeding and bathing, facilitating close contact among individuals. Given that foraging is the primary daily activity of cranes and that they are omnivorous, the most plausible route of infection is cohabitation with infected birds, along with potential transmission via contaminated environments. Such behaviors may promote the spread of the HPAI virus. Infections resulting from this contact can expose birds from other colonies, leading to further dissemination of the virus to new locations and susceptible avian species.

## 5 Conclusion

In summary, these findings demonstrate that common cranes are highly susceptible to natural infection with HPAI H5N1 virus of clade 2.3.4.4b. Neurologic signs were the primary clinical manifestation, while pancreatic lesions were the most common gross finding. Considering that cranes are migratory birds, their seasonal movements could facilitate viral interactions among different bird populations and species, between breeding and wintering grounds, or within habitats. Due to their large size, which makes them easy to locate, and their concentrated habitat distribution, these findings will enhance future virus detection efforts in this species and emphasize the need for extensive surveillance systems in migratory cranes. Therefore, cranes may serve as bio-sentinels for the presence of the HPAI virus in wildlife, with future susceptibility potentially being influenced by the immune status of the population. A number of environmental and climatic factors, including extreme weather events, climate change and habitat destruction, may also directly or indirectly affect the ecology and demography of cranes and thereby influence the future dynamics of HPAI infections. Cranes are often conservation-dependent due to these threats, and this HPAI outbreak exacerbates their vulnerability, making conservation measures even more important. Monitoring and protecting crane populations from HPAI is essential to prevent further population declines.

## Data Availability

The datasets presented in this study can be found in online repository: NCBI GenBank, accession numbers PQ685008 - PQ685015.
