# Supplementary material for: Natural infection of common cranes (Grus grus) with highly pathogenic avian influenza H5N1 in Serbia
Source: Front Vet Sci. 2024 Dec 9;11:1462546. doi: 10.3389/fvets.2024.1462546 (PMC11664861; doi:10.3389/fvets.2024.1462546)
Supplement: Supplementary file 1 [file Table_1.DOCX]

**Table 1.** Frequency and severity of gross and histological lesions in the carcasses of common cranes naturally infected with HPAI H5N1.

| **Tissue** | **Gross pathology** | | **Histopatology** | |
| --- | --- | --- | --- | --- |
|  | **n (%)** | **grade** | **n (%)** | **grade** |
| **Lungs** | 5/14 (36) | ++ to ++++ | 7/14 (50) | ++ to ++++ |
| **Spleen** | 6/14 (43) | ++ to +++ | 7/14 (50) | ++ to +++ |
| **Liver** | 4/14 (28) | + to +++ | 7/14 (50) | +++ |
| **Kidney** | 5/14 (36) | + to ++ | 5/14 (36) | ++ |
| **Pancreas** | 14/14 (100) | ++++ | 14/14 (100) | ++++ |
| **Brain** | 3/14 (21) | ++ | 9/14 (64) | +++ |
| **Heart** | 1/14 (7) | + | / | / |
| **Intestine** | 0/14 (0) | - | / | / |
| **Gizzard** | 0/14 (0) | - | / | / |

Gross and histopathology grade: Absent -, minimal +, mild ++, moderate +++, severe ++++

/ - not performed
